# Supplementary material for: Biomimetic Red Blood Cell Membrane-Mediated Nanodrugs Loading Ursolic Acid for Targeting NSCLC Therapy
Source: Cancers (Basel). 2022 Sep 18;14(18):4520. doi: 10.3390/cancers14184520 (PMC9496832; doi:10.3390/cancers14184520)
Supplement: Supplementary file 1 [file cancers-14-04520-s001.zip › cancers-1855416-supplementary.pdf]

cancers

Supplementary Materials: Biomimetic Red Blood Cell Membranes-Mediated Nanodrugs Loading Ursolic Acid for Targeting NSCLC Therapy

Ting Wu, Dan Yan, Wenjun Hou, Hui Jiang, Min Wu, Yanling Wang, Gang Chen, Chunming Tang, Yijun Wang and Huae Xu

Table S1. The size and zeta-potential of UaNPs (a) and UMNPs (b).

Samples	Size(nm)	Zeta-potential(mV)
UaNPs	100.5±1.05%	-17.23±0.16%
UMNPs	112.0±2.96%	-24.47±0.52%

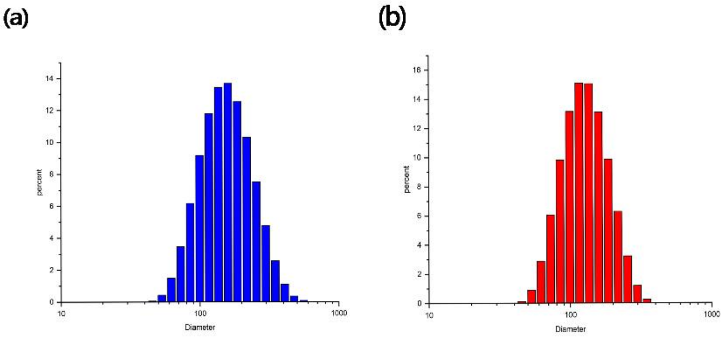

Figure S1. The size of UaNPs (a) and UMNPs (b).

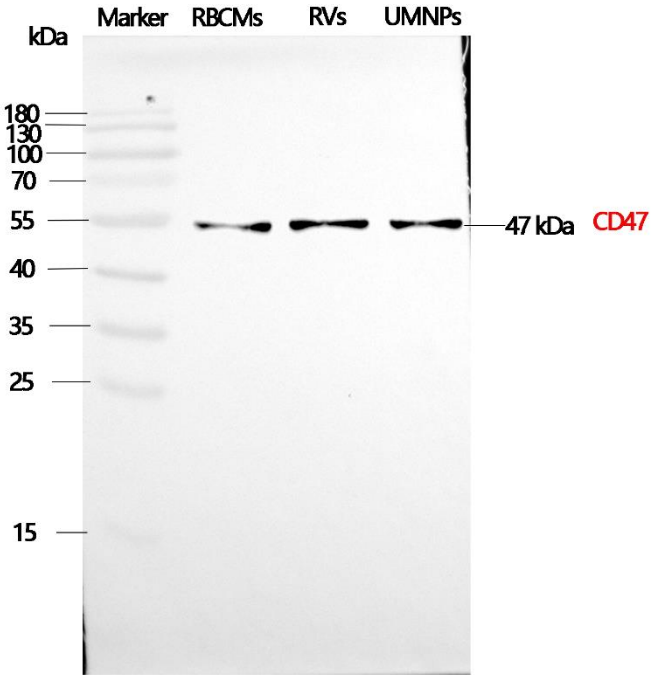

Figure S2. Original western blots picture of CD47.

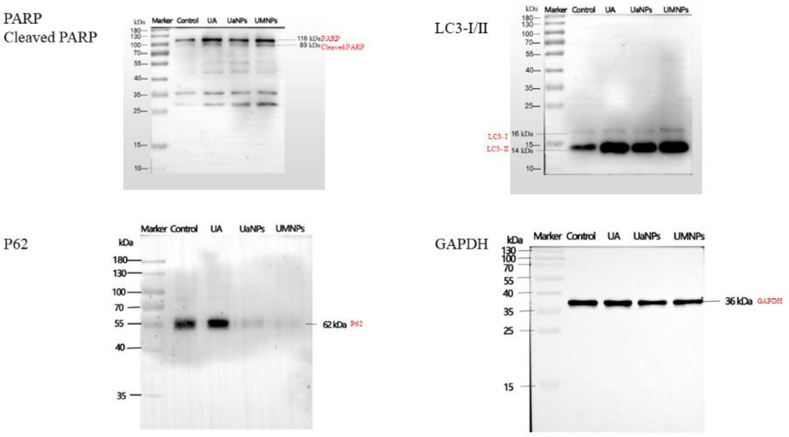

Figure S3. Original western blots picture of PARP LC3 P62.

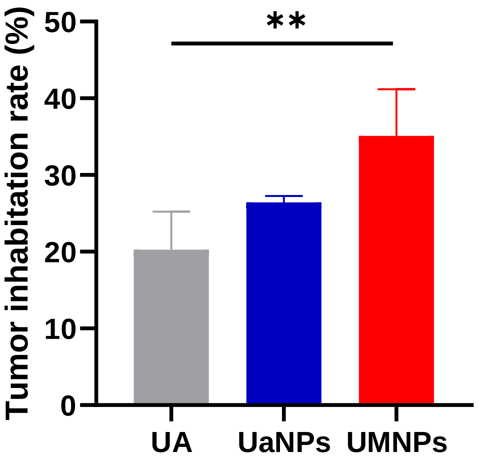

Figure S4. TIR of UaNPs and UMNPs. Differences were considered significant when $^{**}p < 0.01$.

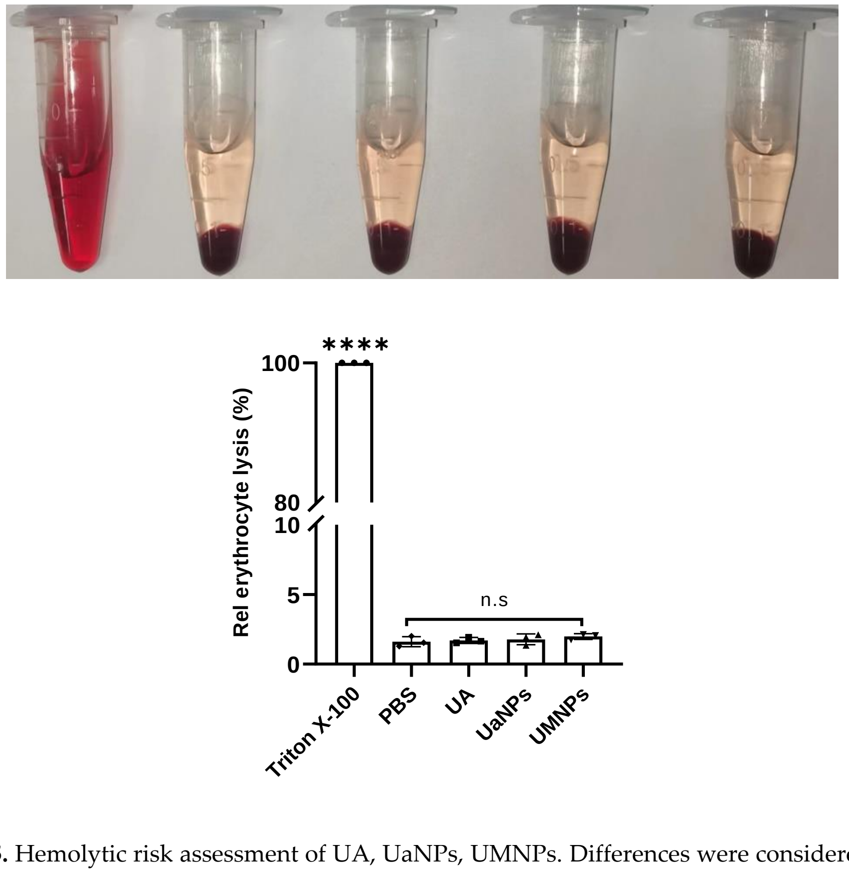

Figure S5. Hemolytic risk assessment of UA, UaNPs, UMNPs. Differences were considered signifi-cant when $^{****}p < 0.0001$, while there is no significant when $p > 0.05$ which means ns.
